# Supplementary material for: Adverse cardiovascular events and cardiac imaging findings in patients on immune checkpoint inhibitors
Source: PLoS One. 2024 Dec 2;19(12):e0314555. doi: 10.1371/journal.pone.0314555 (PMC11611253; doi:10.1371/journal.pone.0314555)
Supplement: S2 Fig — (DOCX) [file pone.0314555.s002.docx]

|  |  |
| --- | --- |
| **Variable** | **Median Time to Adverse Outcome [Days (IQR)]** |
| ACE | 127 (42, 296) |
| ASCVD | 175 (64, 370) |
| Arrhythmia | 132 (43, 311) |
| Heart failure | 183 (64, 446) |
| Myocarditis | 220 (65, 322) |
| Pericardial disease | 117 (28, 270) |
| Valvular disease | 252 (93, 495) |


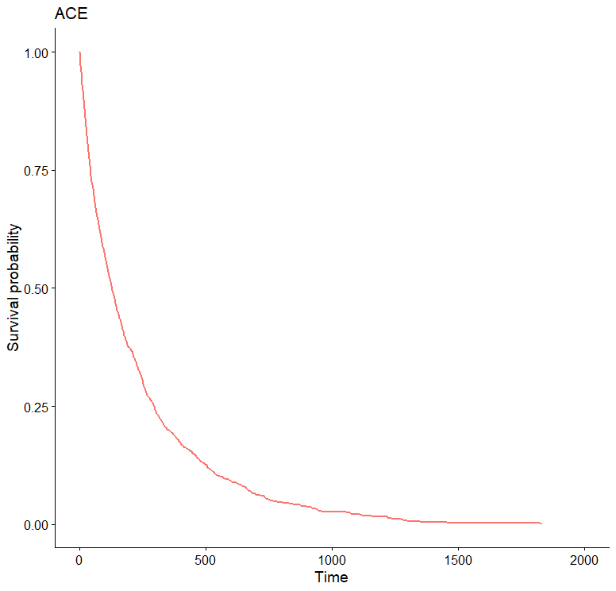

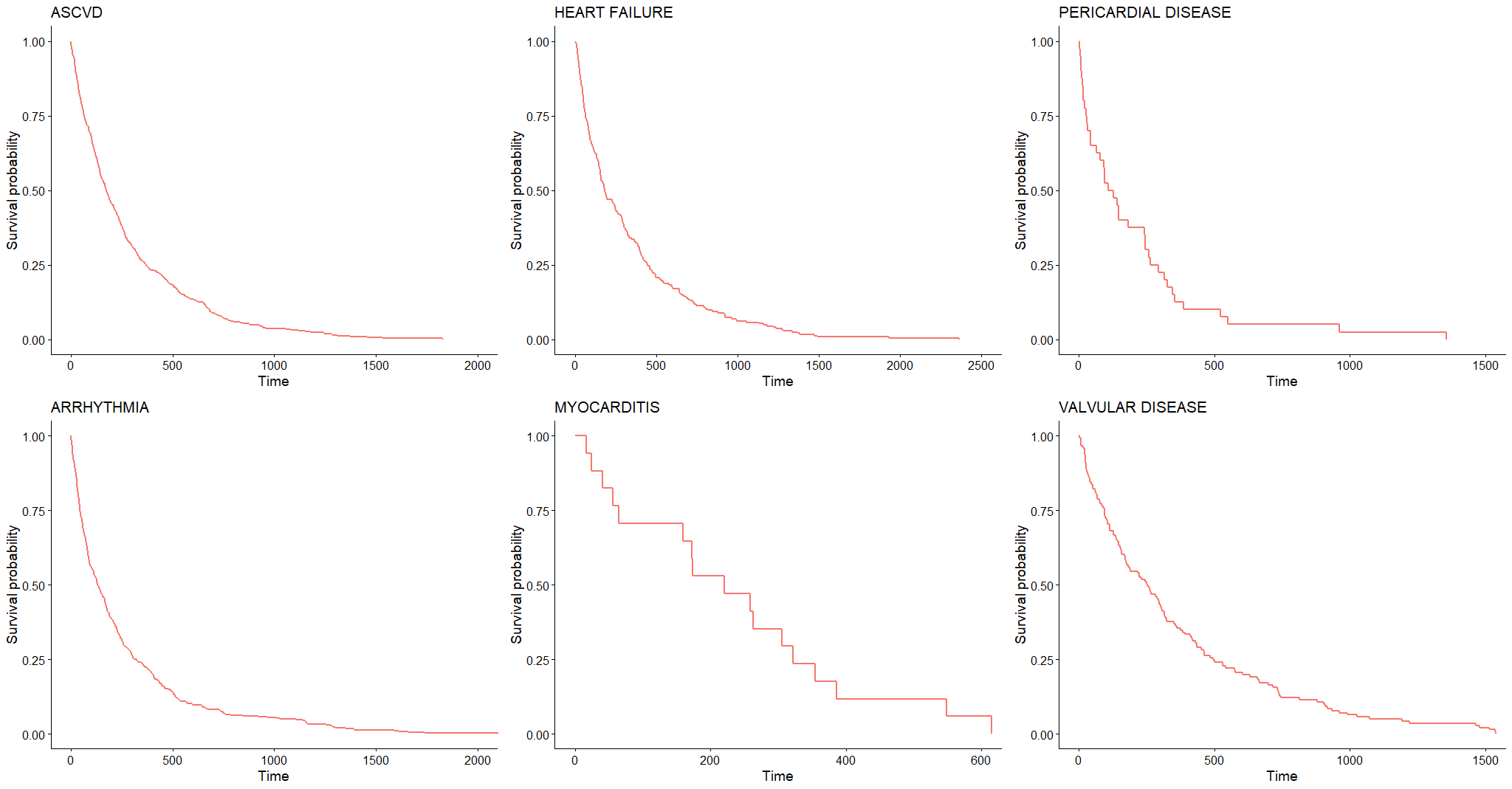


**SUPPLEMENTAL FIGURE 2. KAPLAN MEIER CURVES AND MEDIAN TIMES TO ADVERSE CARDIOVASCULAR EVENTS (ACE) AND INDIVIDUAL COMPONENTS OF ACE**
