# Supplementary material for: Adverse cardiovascular events and cardiac imaging findings in patients on immune checkpoint inhibitors
Source: PLoS One. 2024 Dec 2;19(12):e0314555. doi: 10.1371/journal.pone.0314555 (PMC11611253; doi:10.1371/journal.pone.0314555)
Supplement: S3 Fig — (DOCX) [file pone.0314555.s003.docx]

**SUPPLEMENTAL FIGURE 3. COX REGRESSION MODEL INCLUDING SINGLE IMMUNE CHECKPOINT INHIBITOR AGENTS FOR ADVERSE CARDIOVASCULAR EVENTS (ACE) AND INDIVIDUAL COMPONENTS OF ACE**


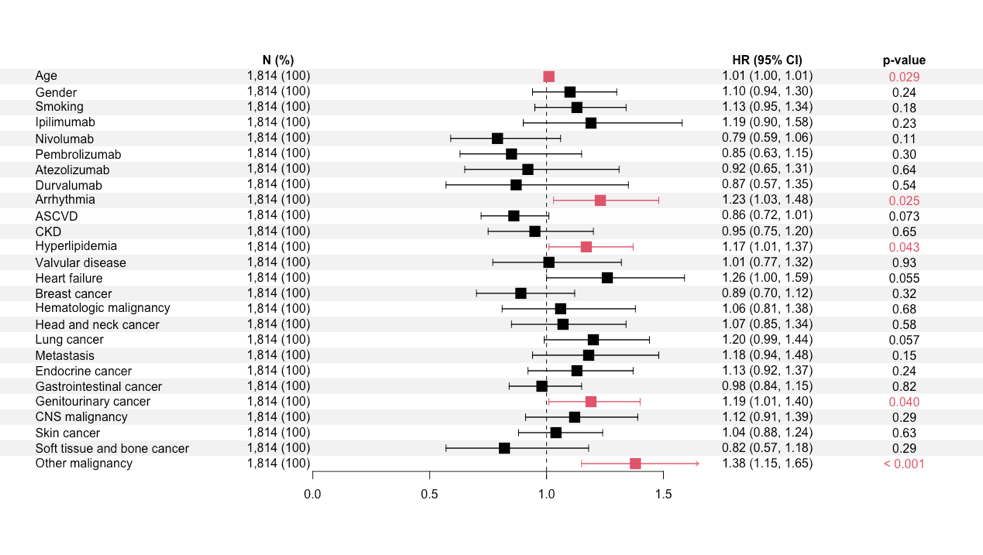

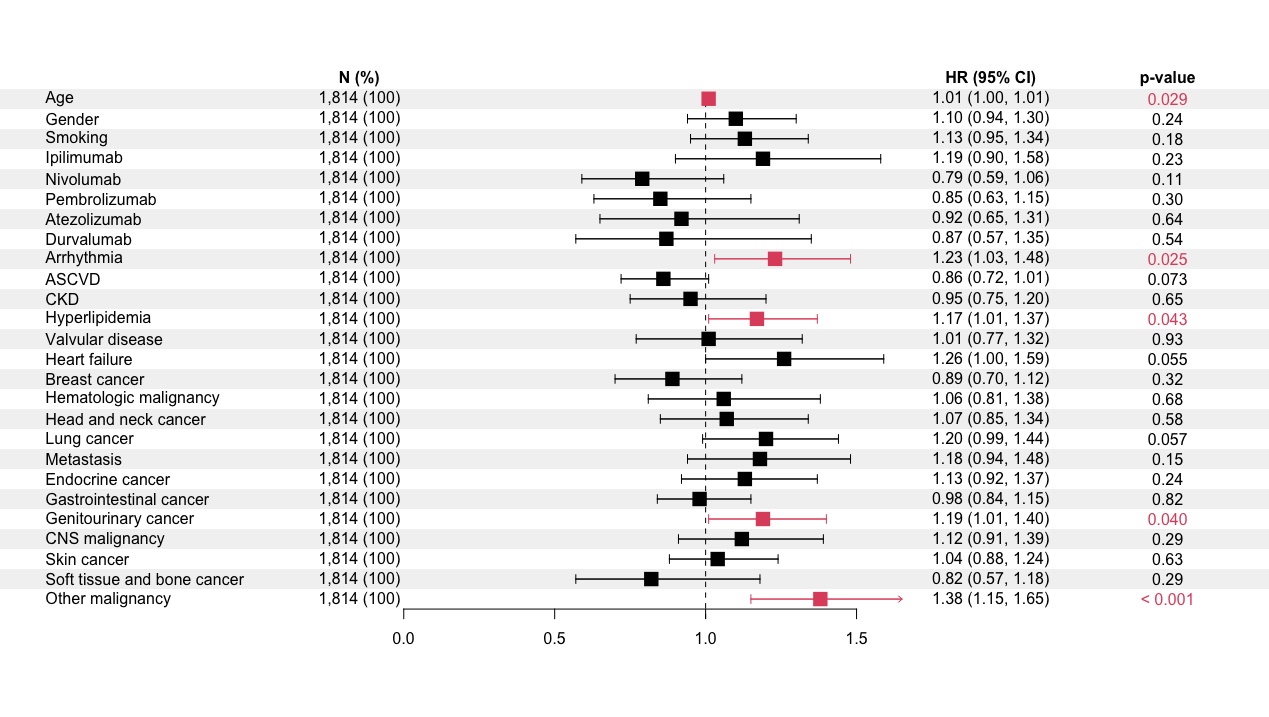

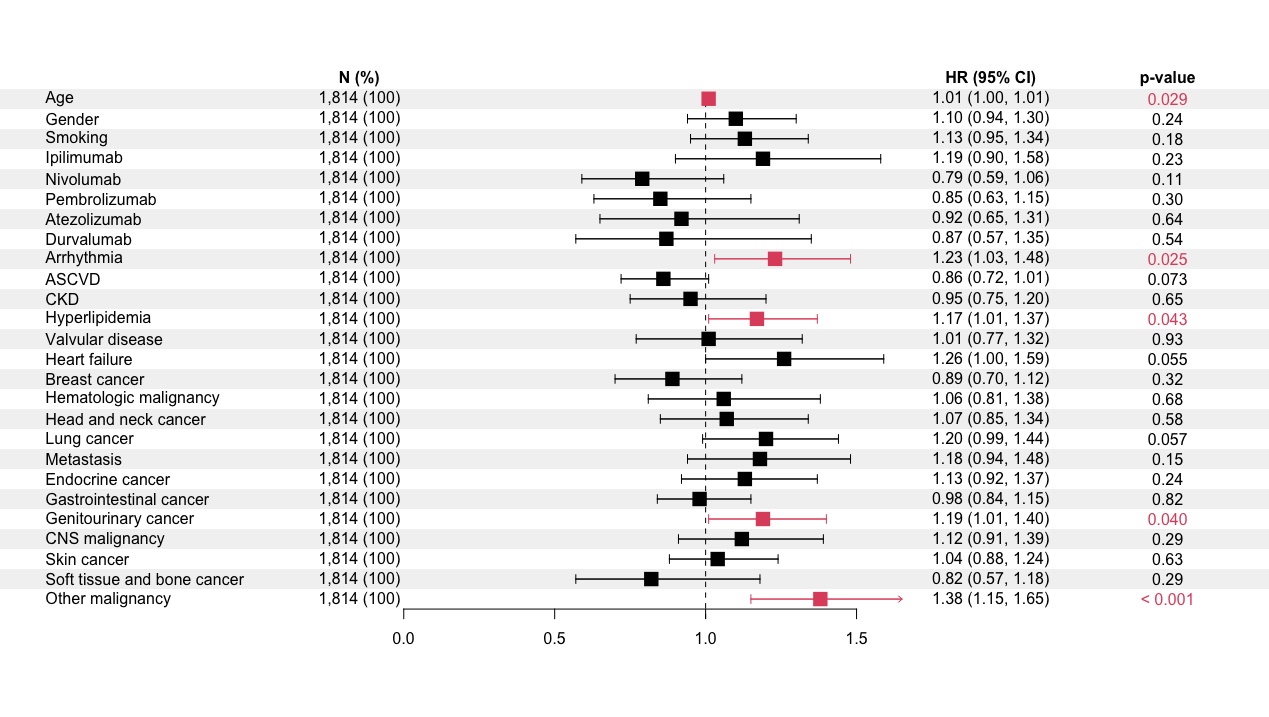


**A) ACE**


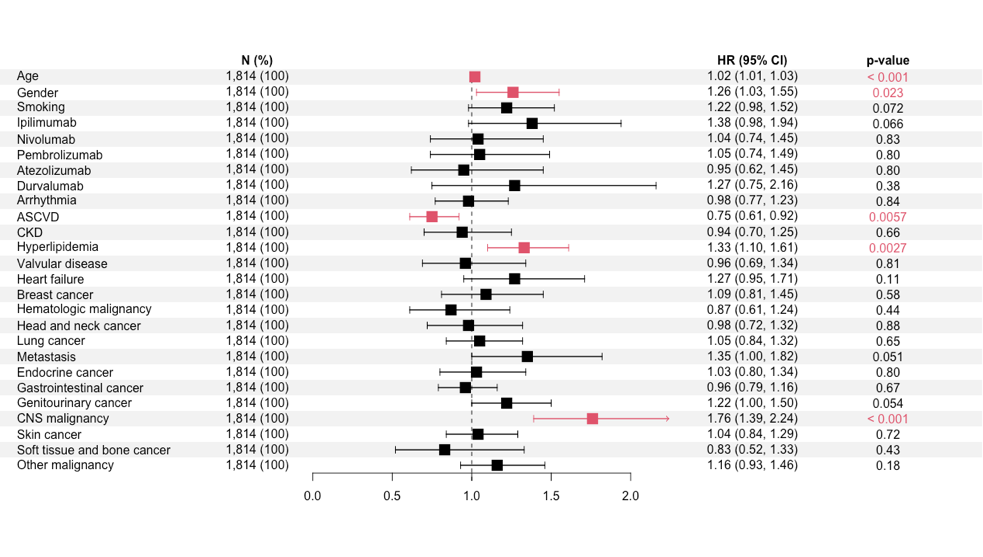

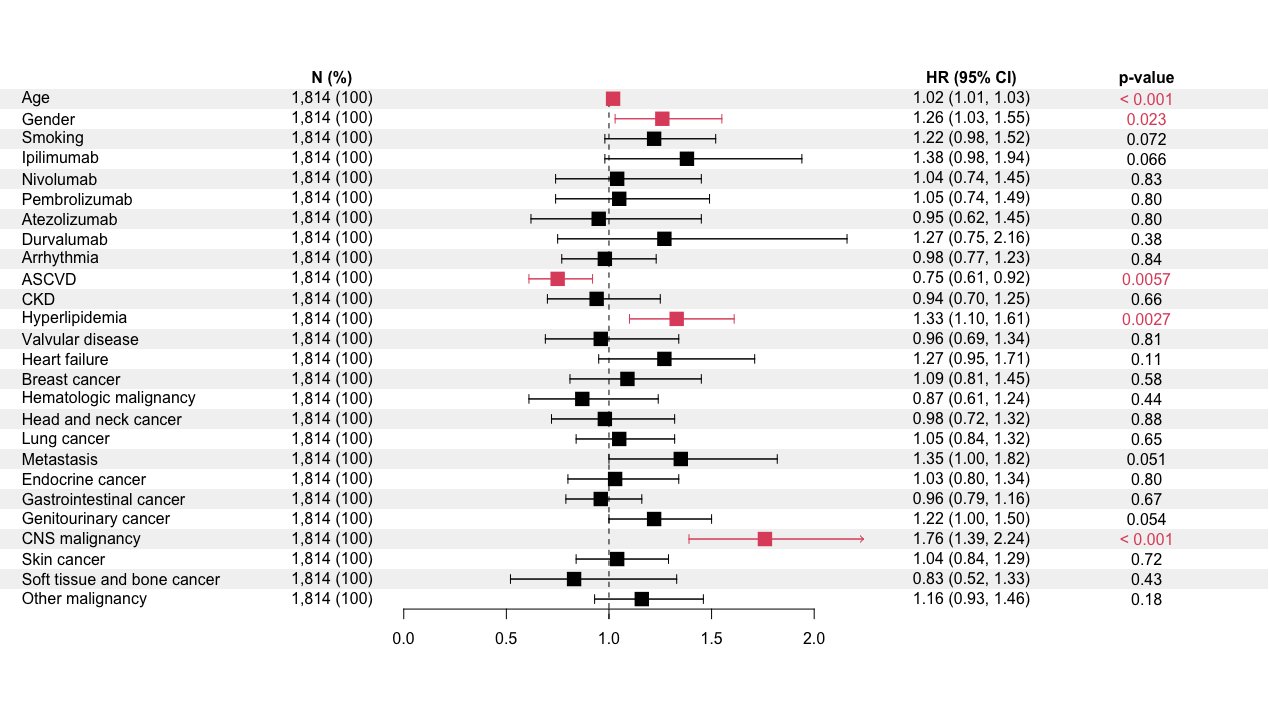

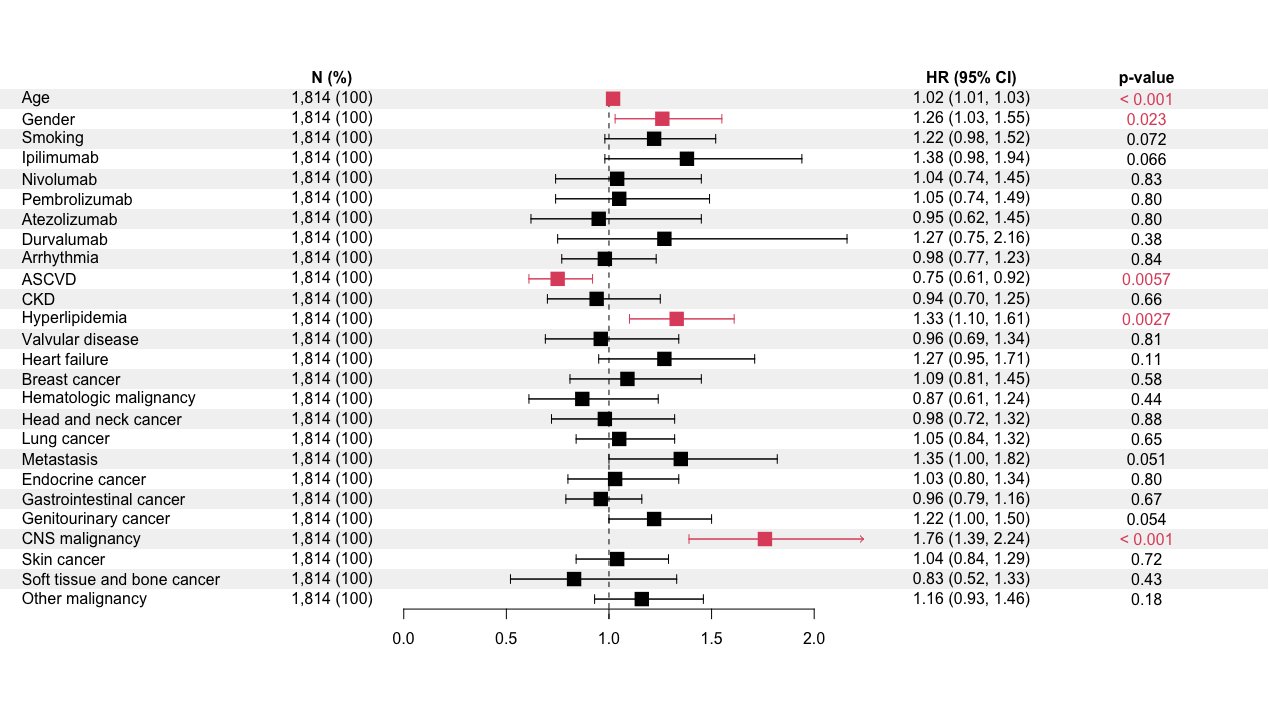


**B) ASCVD**


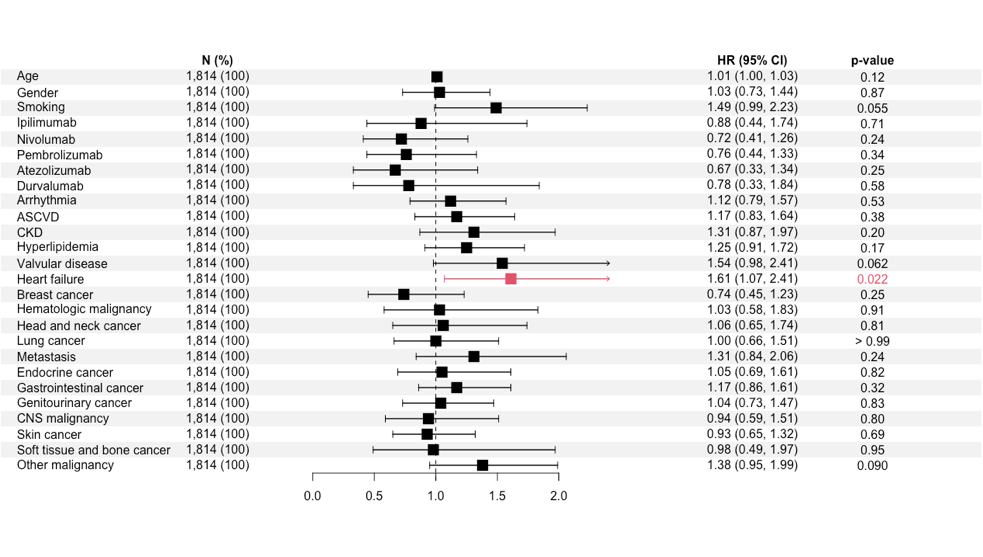

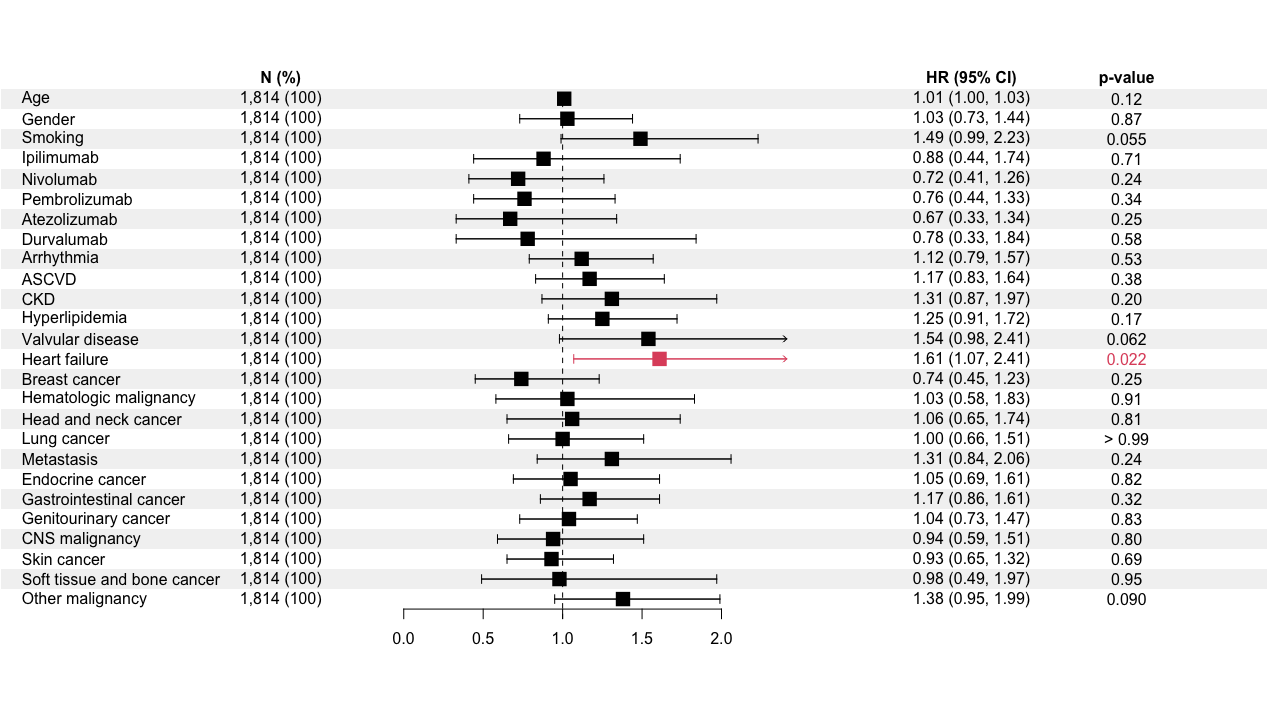

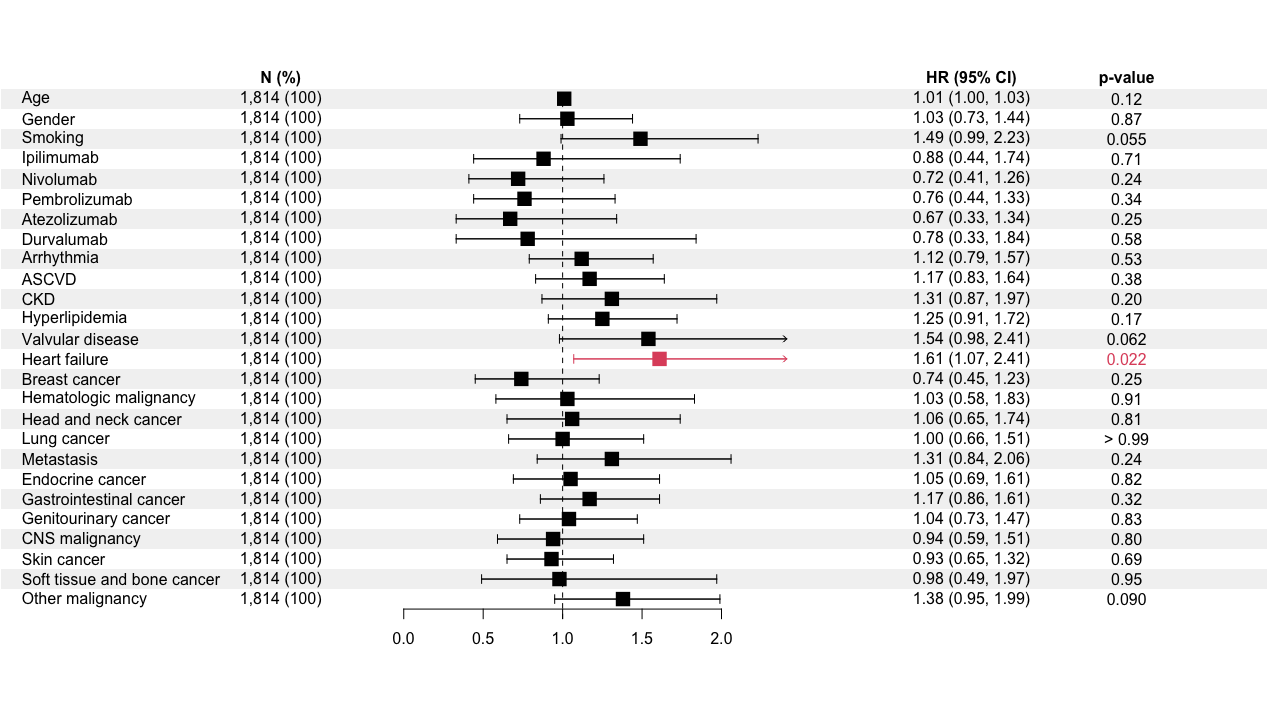


**C) Heart Failure**


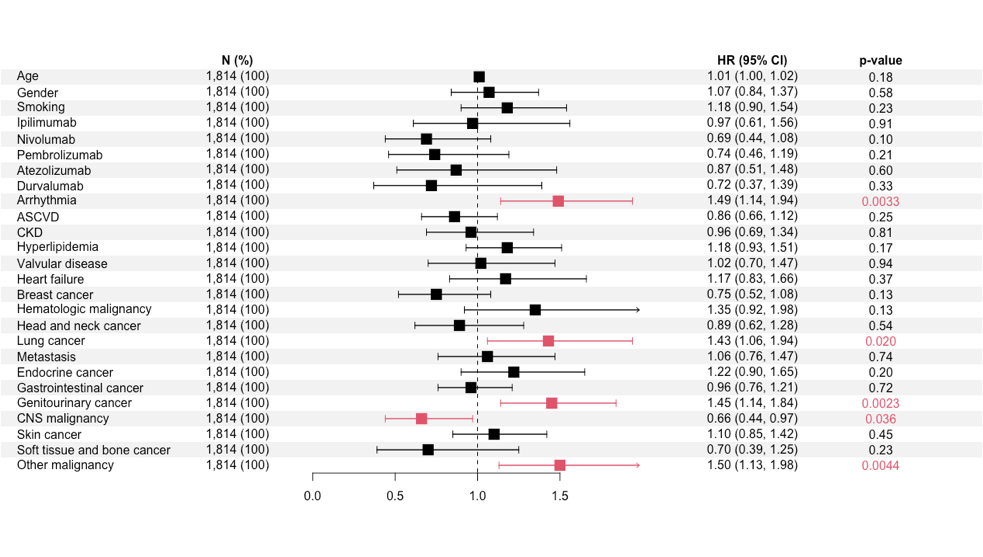

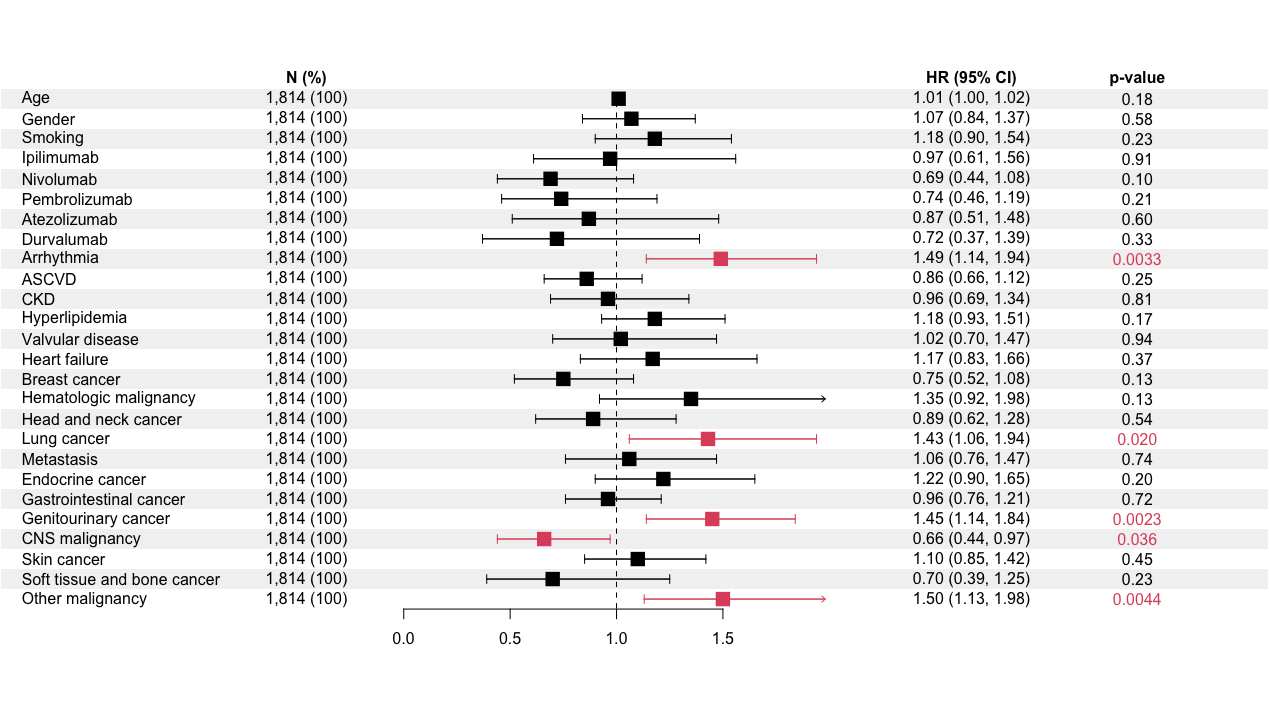

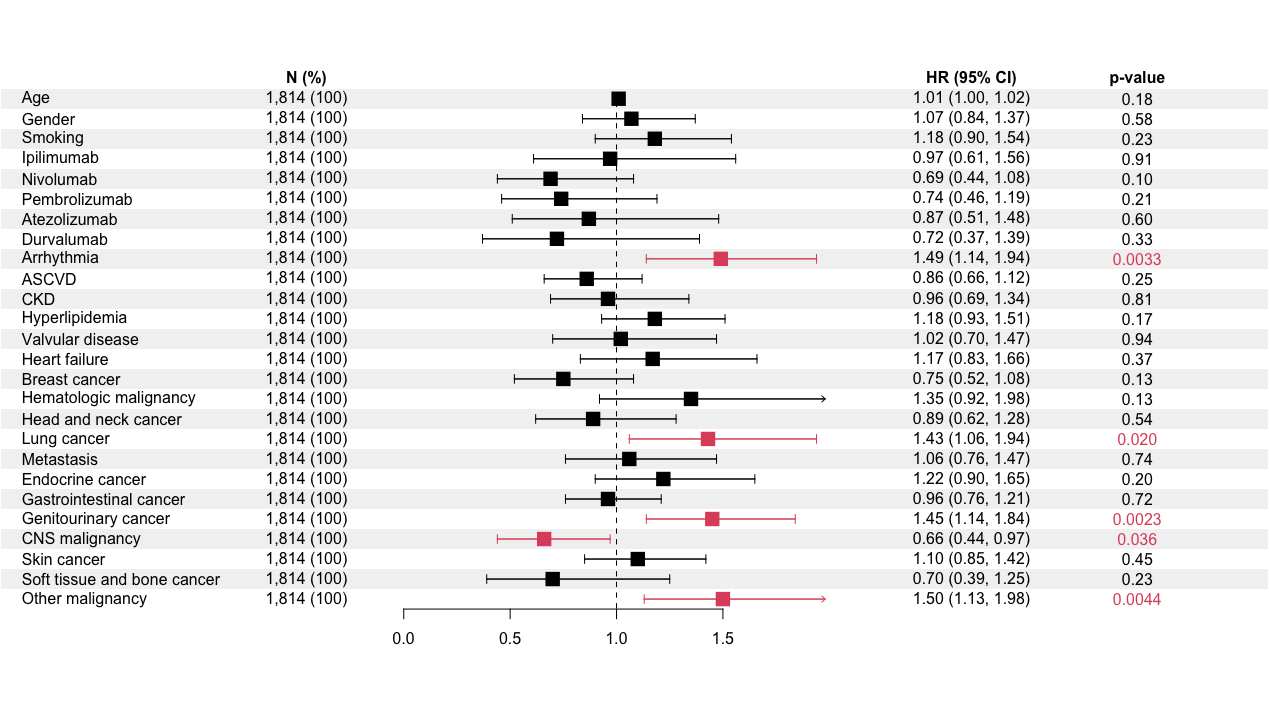


**D) Arrhythmia**


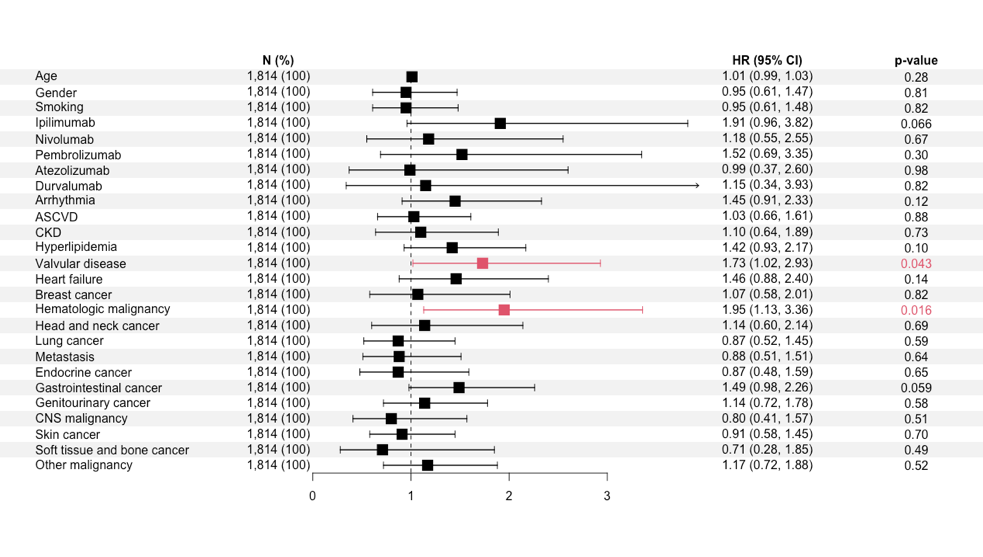

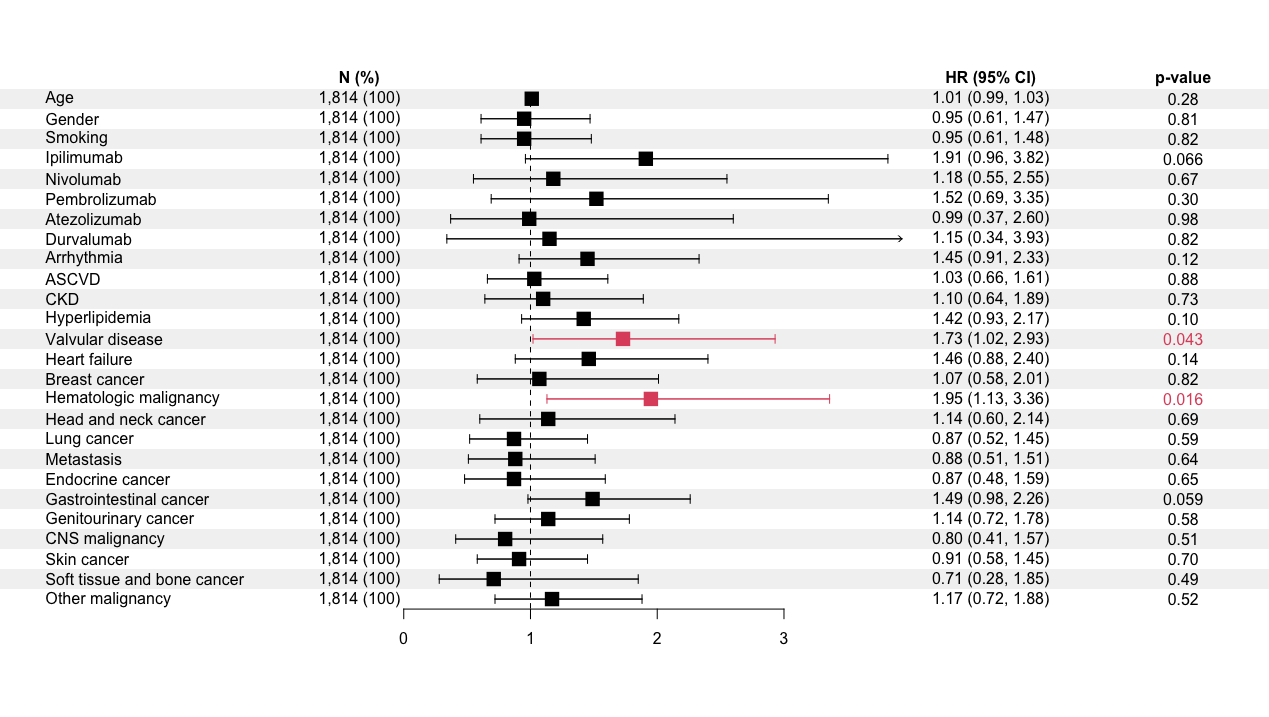

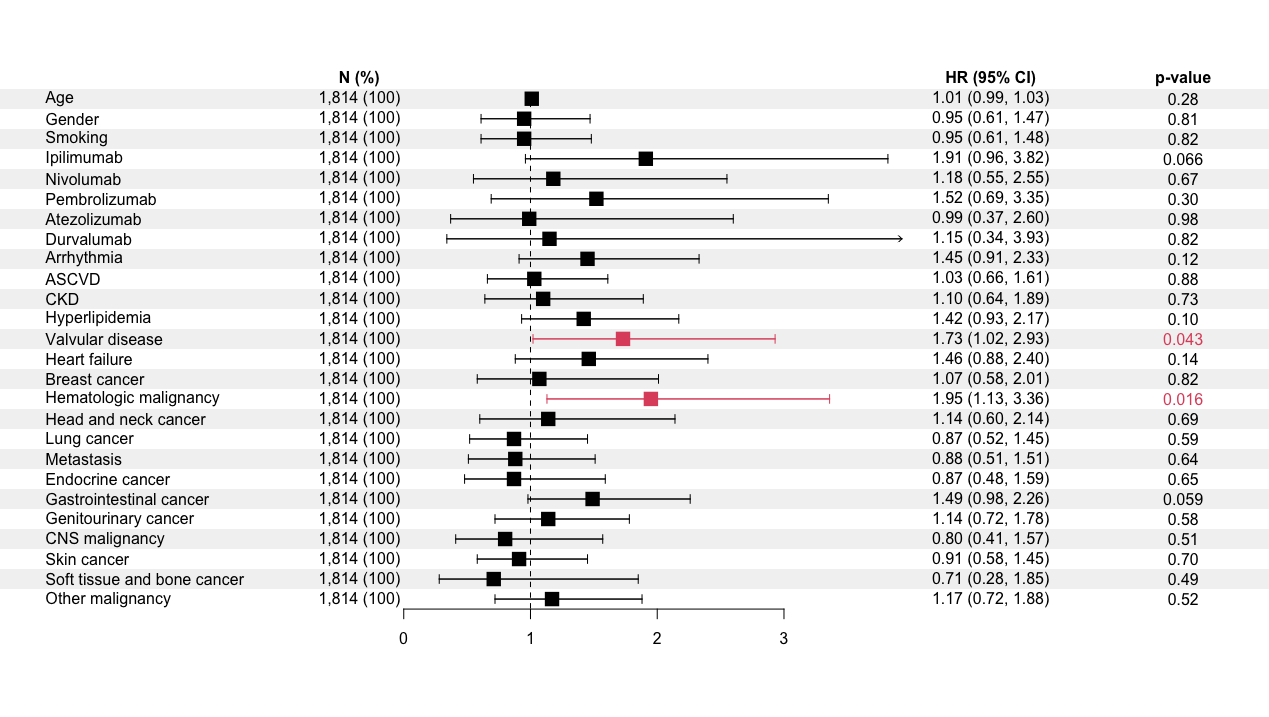

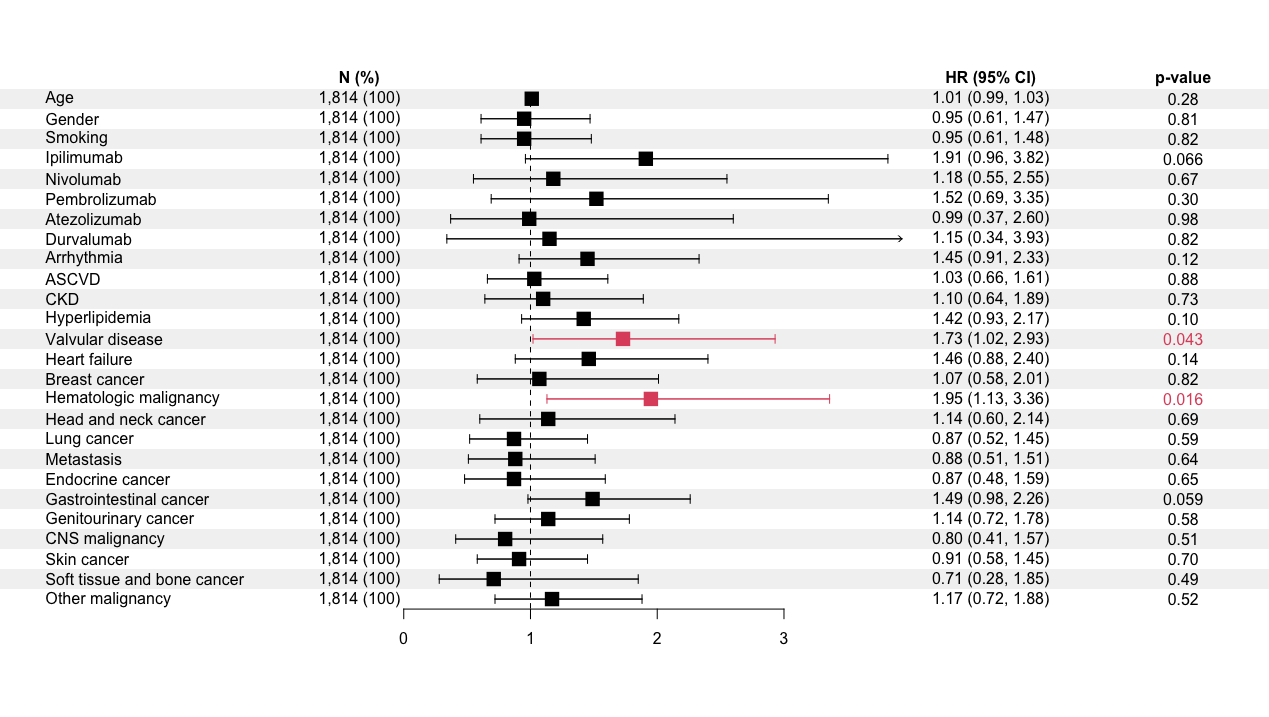


**E) Valvular Disease**
