# Supplementary material for: Adverse cardiovascular events and cardiac imaging findings in patients on immune checkpoint inhibitors
Source: PLoS One. 2024 Dec 2;19(12):e0314555. doi: 10.1371/journal.pone.0314555 (PMC11611253; doi:10.1371/journal.pone.0314555)

**SUPPLEMENTAL FIGURE 4.** **PREVALENCE OF ABNORMAL ECHOCARDIOGRAMS PRE- AND POST-IMMUNE CHECKPOINT INHIBITOR**


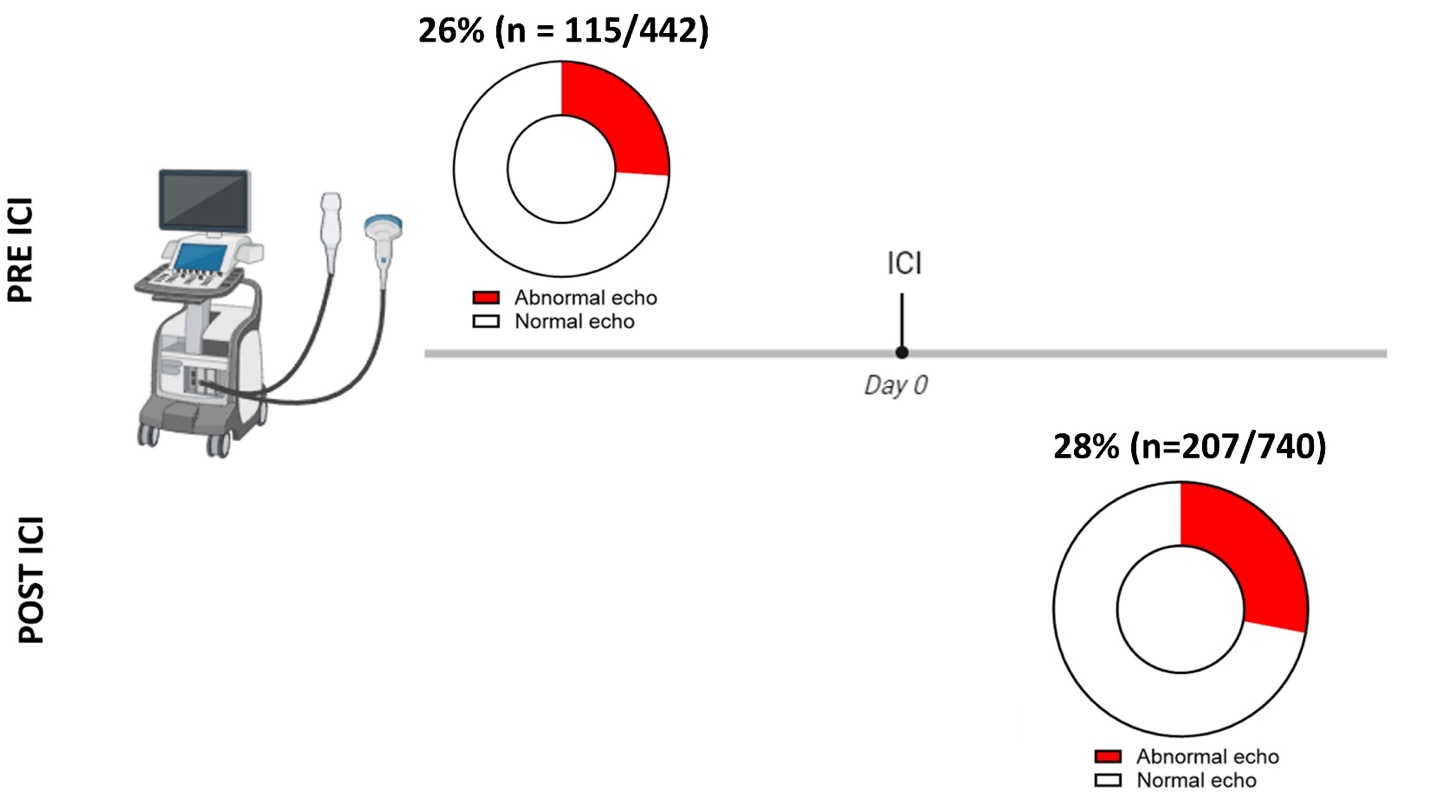

Supplement: S4 Fig — (DOCX) [file pone.0314555.s004.docx]
