# Supplementary material for: Adverse cardiovascular events and cardiac imaging findings in patients on immune checkpoint inhibitors
Source: PLoS One. 2024 Dec 2;19(12):e0314555. doi: 10.1371/journal.pone.0314555 (PMC11611253; doi:10.1371/journal.pone.0314555)
Supplement: S1 Table — (DOCX) [file pone.0314555.s005.docx]

**SUPPLEMENTAL TABLE 1. COMPARISON OF DEMOGRAPHIC CHARACTERISTICS AND COMORBIDITIES BETWEEN ENTIRE COHORT FROM ELECTRONIC HEALTH RECORD DATA ACQUISITION AND ADJUDICATED SUBSET**

| **Variable** | **Subset Total** | ***P*-value (vs total cohort)** |
| --- | --- | --- |
| Age (mean, SD) | 68, 11.49 | 0.297 |
| Female | 43% (86/200) | 0.882 |
| Arrhythmia | 21% (42/200) | 0.538 |
| ASCVD | 36% (72/200) | 0.5917 |
| Heart failure | 6% (12/200) | 0.062 |
| Hypertension | 69.5% (139/200) | 0.104 |
| Hyperlipidemia | 57% (114/200) | 0.00002 |
| Diabetes mellitus | 22.5% (45/200) | 0.718 |
| Chronic kidney disease | 10.5% (21/200) | 0.4534 |
