# Supplementary material for: Adverse cardiovascular events and cardiac imaging findings in patients on immune checkpoint inhibitors
Source: PLoS One. 2024 Dec 2;19(12):e0314555. doi: 10.1371/journal.pone.0314555 (PMC11611253; doi:10.1371/journal.pone.0314555)
Supplement: S2 Table — (DOCX) [file pone.0314555.s006.docx]

**SUPPLEMENTAL TABLE 2. PRESENCE OF ADVERSE CARDIOVASCULAR EVENTS BY CANCER TYPE**

| **Cancer Type** | **No ACE** | **ACE** | **P-value** |
| --- | --- | --- | --- |
| Lung cancer | 54.3% (846/1558) | 46.4% (712/1558) | 0.075 |
| Melanoma | 53.3% (333/625) | 46.7% (292/625) | 0.182 |
| Liver cancer | 55% (298/542) | 45% (244/542) | 0.803 |
| Gastrointestinal cancer | 56% (187/334) | 44% (147/334) | 0.858 |
| Breast cancer | 60.9% (159/258) | 38.4% (99/258) | **0.038** |
| Head and neck cancer | 53% (116/219) | 47% (103/219) | 0.431 |
| CNS malignancy | 46.7% (98/210) | 53.3% (112/210) | **0.007** |
| Renal cancer | 52.2% (105/201) | 47.8% (96/201) | 0.333 |
| Prostate cancer | 48.5% (97/200) | 51.5% (103/200) | **0.037** |
| Hematologic malignancy | 47.8% (77/161) | 52.2% (84/161) | **0.048** |
| Bladder cancer | 49.7% (78/157) | 50.3% (79/157) | 0.340 |
| Pancreatic cancer | 47.5% (58/122) | 52.5% (64/122) | 0.075 |
| Neuroendocrine tumors | 52.3% (45/86) | 47.7% (41/86) | 0.580 |
| Bone cancer | 52.9% (36/68) | 47.1% (32/68) | 0.711 |
| Ovarian cancer | 52.3% (23/44) | 47.7% (21/44) | 0.760 |
| Cervical cancer | 51.2% (22/43) | 48.8% (21/43) | 0.643 |
| Spine tumors | 46.5% (20/43) | 53.5% (23/43) | 0.278 |
| Thyroid cancer | 50% (21/42) | 50% (21/42) | 0.531 |
| Adrenal cancer | 52.6% (20/38) | 47.4% (18/38) | 0.744 |
| Uterine cancer | 51.4% (18/35) | 48.6% (17/35) | 0.732 |
| Carcinoid tumor | 41.9% (13/31) | 58.1% (18/31) | 0.146 |
| Endometrial cancer | 48.1% (13/27) | 51.9% (14/27) | 0.444 |
| Sarcoma | 61.9.% (13/21) | 38.1% (8/21) | 0.661 |
| Vulvar cancer | 68.4% (13/19) | 31.6% (6/19) | 0.354 |
| Eye tumors | 47.1% (8/17) | 52.9% (9/17) | 0.625 |
| Cardiac tumors | 85.7% (12/14) | 14.3% (2/14) | **0.028** |
| Pituitary cancer | 66.7% (6/9) | 33.3% (3/9) | 0.739 |
| Gallbladder cancer | 50% (3/6) | 50% (3/6) | 1.000 |
| Endocrine cancer | 33.3% (1/3) | 66.7% (2/3) | 0.588 |
| Parotid cancer | 0% (0/3) | 100% (3/3) | 0.088 |
| Testicular cancer | 100% (3/3) | 0% (0/3) | 0.259 |
| Soft tissue cancer | 100% (2/2) | 0% (0/2) | 0.506 |
| Other malignancy | 47.6% (127/267) | 52.4% (140/267) | **0.006** |
| Metastatic disease | 54.5% (1018/1869) | 45.5% (851/1869) | **0.014** |
