# Supplementary material for: Adverse cardiovascular events and cardiac imaging findings in patients on immune checkpoint inhibitors
Source: PLoS One. 2024 Dec 2;19(12):e0314555. doi: 10.1371/journal.pone.0314555 (PMC11611253; doi:10.1371/journal.pone.0314555)
Supplement: S3 Table — (DOCX) [file pone.0314555.s007.docx]

| **SUPPLEMENTAL TABLE 3. DEMOGRAPHICS AND COMORBIDITIES OF PROPENSITY MATCHED COHORT** | | | | |
| --- | --- | --- | --- | --- |
|  | **Total (N = 1518)** | **ICI (N = 759)** | **No ICI (N = 759)** | ***P*-value** |
| **Demographic Characteristics** | | | | |
| Age - Mean ± SD (years) | 70.06 ± 12.40 | 69.9 ± 11.7 | 70.2 ± 13.1 | 0.707 |
| BMI - Mean ± SD (kg/m^2^) | 26.71 ± 4.06 | 26.9 ± 3.92 | 26.5 ± 4.20 | 0.039 |
| Male - % (n/N) | 47.8% (725/1518) | 49% (372/759) | 46.5% (353/759) | 0.355 |
| Hispanic - % (n/N) | 4.7% (70/1495) | 3.9% (29/736) | 5.4% (41/759) | <0.001 |
| Race - % (n/N) |  |  |  | <0.001 |
| White | 82.1% (1247/1518) | 85.5% (649/759) | 78.8% (598/759) |  |
| Black | 7.8% (119/1518) | 5.8% (44/759) | 9.9% (75/759) |  |
| Asian | 2.4% (37/1518) | 1.3% (10/759) | 3.6% (27/759) |  |
| Other | 7.6% (115/1518) | 7.4% (56/759) | 7.8% (59/759) |  |
| **Comorbidities - % (n/N)** | | | | |
| Arrhythmia | 22.2% (337/1518) | 22% (167/759) | 22.4% (170/759) | 0.902 |
| ASCVD | 31% (471/1518) | 31.5% (239/759) | 30.6% (232/759) | 0.739 |
| Heart failure | 10.5% (159/1518) | 10.7% (81/759) | 10.3% (78/759) | 0.867 |
| Valvular disease | 8% (122/1518) | 8% (61/759) | 8% (61/759) | 1.000 |
| Hyperlipidemia | 32% (486/1518) | 31.5% (239/759) | 32.5% (247/759) | 0.700 |
| Ever smoker | 68.6% (1039/1514) | 78.7% (596/757) | 58.5% (443/757) | <0.001 |
| Chronic kidney disease | 12.8% (195/1518) | 12.5% (95/759) | 13.2% (100/759) | 0.759 |
| Metastatic disease | 80% (1214/1518) | 80.6% (612/759) | 79.3% (602/759) | 0.564 |
| **Cancer type** | | | | |
| GI cancer | 29.8% (452/1518) | 19.5% (148/759) | 40.1% (304/759) | <0.001 |
| Lung cancer | 75.8% (1150/1518) | 90.4% (686/759) | 61.1% (464/759) | <0.001 |
| Renal cancer | 12.5% (189/1518) | 10.1% (77/759) | 14.8% (112/759) | 0.008 |
