# Supplementary material for: Adverse cardiovascular events and cardiac imaging findings in patients on immune checkpoint inhibitors
Source: PLoS One. 2024 Dec 2;19(12):e0314555. doi: 10.1371/journal.pone.0314555 (PMC11611253; doi:10.1371/journal.pone.0314555)
Supplement: S4 Table — (DOCX) [file pone.0314555.s008.docx]

**SUPPLEMENTAL TABLE 4. ORDERING INDICATIONS FOR CARDIAC MAGNETIC RESONANCE IMAGING PRE- AND POST-IMMUNE CHECKPOINT INHIBITOR**

| Indication | Pre-ICI (n [%]) | | Post-ICI (n [%]) | |
| --- | --- | --- | --- | --- |
| Oncology therapy toxicity | 2 | (7.14) | 53 | (51.96) |
| Cardiomyopathy/troponin elevated | 6 | (21.43) | 19 | (18.63) |
| Chest pain/shortness of breath | 0 | (0.00) | 1 | (0.98) |
| Arrhythmia/abnormal ECG | 0 | (0.00) | 0 | (0.00) |
| Mass/metastasis | 8 | (28.57) | 18 | (17.65) |
| Thrombus | 6 | (21.43) | 3 | (2.94) |
| Structural evaluation | 5 | (17.86) | 6 | (5.88) |
| Pericarditis | 1 | (3.57) | 2 | (1.96) |
| Total | **28** |  | **102** |  |
