# Supplementary material for: Adverse cardiovascular events and cardiac imaging findings in patients on immune checkpoint inhibitors
Source: PLoS One. 2024 Dec 2;19(12):e0314555. doi: 10.1371/journal.pone.0314555 (PMC11611253; doi:10.1371/journal.pone.0314555)
Supplement: S5 Table — (DOCX) [file pone.0314555.s009.docx]

**SUPPLEMENTAL TABLE 5. CARDIAC MAGNETIC RESONANCE IMAGING FEATURES**

**A)**

| LVEF (%) |  | Mean | Median | Q1 | Q3 | *P-*value |
| --- | --- | --- | --- | --- | --- | --- |
|  | **Pre-ICI** | 53.9 | 58 | 47.5 | 62 | 0.283 |
|  | **Post-ICI** | 48.7 | 49 | 43.2 | 59.8 |  |
|  | **Total** | 53.1 | 57 | 47 | 61 |  |
| Cardiac Output (L/min) |  |  |  |  |  |  |
|  | **Pre-ICI** | 5.7 | 5.3 | 4.2 | 6.8 | 0.071 |
|  | **Post-ICI** | 5.06 | 4.76 | 4.54 | 5.85 |  |
|  | **Total** | 5.56 | 5.12 | 4.2 | 6.5 |  |
| Cardiac Index (L/min/m^2^) |  |  |  |  |  |  |
|  | **Pre-ICI** | 3.01 | 2.9 | 2.4 | 3.32 | **0.024** |
|  | **Post-ICI** | 2.64 | 2.8 | 2.25 | 3 |  |
|  | **Total** | 2.93 | 2.9 | 2.36 | 3.1 |  |

**B)**

|  | Pre-ICI (% [n/N])  N = 21 | Post-ICI (% [n/N])  N = 83 | Total (% [n/N])  N = 104 | *P*-value |
| --- | --- | --- | --- | --- |
| Any LV LGE* | 31.6% (6/19) | 29.6% (21/71) | 30.0% (27/90) | >0.999 |
| Non-ischemic LV LGE | 7.1% (1/14) | 14.5% (9/62) | 13.2% (10/76) | 0.678 |
| RV insertion LGE | 5.3% (1/19) | 11.3% (8/71) | 10.0% (9/90) | 0.678 |
| Abnormal T2 | 9.1% (1/11) | 12.1% (7/58) | 11.6% (8/69) | >0.999 |
| Reduced LVEF <57% | 42.9% (9/21) | 45.8% (33/72) | 45.2% (42/93) | >0.999 |
| Reduced RVEF <52% | 50.0% (10/20) | 55.1% (38/69) | 53.9% (48/89) | 0.800 |

**C)**

|  | No ICI Myocarditis (% [n/N])  N = 90 | ICI Myocarditis (% [n/N])  N = 14 | Total (% [n/N])  N = 104 | *P*-value |
| --- | --- | --- | --- | --- |
| Any LV LGE* | 24.7% (19/77) | 61.5% (8/13) | 30.0% (27/90) | **0.018** |
| Non-ischemic LV LGE | 7.7% (5/65) | 45.5% (5/11) | 13.2% (10/76) | **0.004** |
| RV insertion LGE | 5.2% (4/77) | 38.5% (5/13) | 10.0% (9/90) | **0.003** |
| Abnormal T2 | 8.3% (5/60) | 33.3% (3/9) | 11.6% (8/69) | 0.063 |
| Reduced LVEF <57% | 43.0% (34/79) | 57.1% (8/14) | 45.2% (42/93) | 0.390 |
| Reduced RVEF <52% | 50.0% (38/76) | 76.9% (10/13) | 53.9% (48/89) | 0.130 |

*Any LV LGE: non-ischemic LV LGE, ischemic LV LGE, or both
