# Supplementary material for: Adverse cardiovascular events and cardiac imaging findings in patients on immune checkpoint inhibitors
Source: PLoS One. 2024 Dec 2;19(12):e0314555. doi: 10.1371/journal.pone.0314555 (PMC11611253; doi:10.1371/journal.pone.0314555)

**Central Illustration.** Risk Factors Increasing Adverse Cardiovascular Events and Cardiac Imaging findings in Patients Pre- and Post-ICI


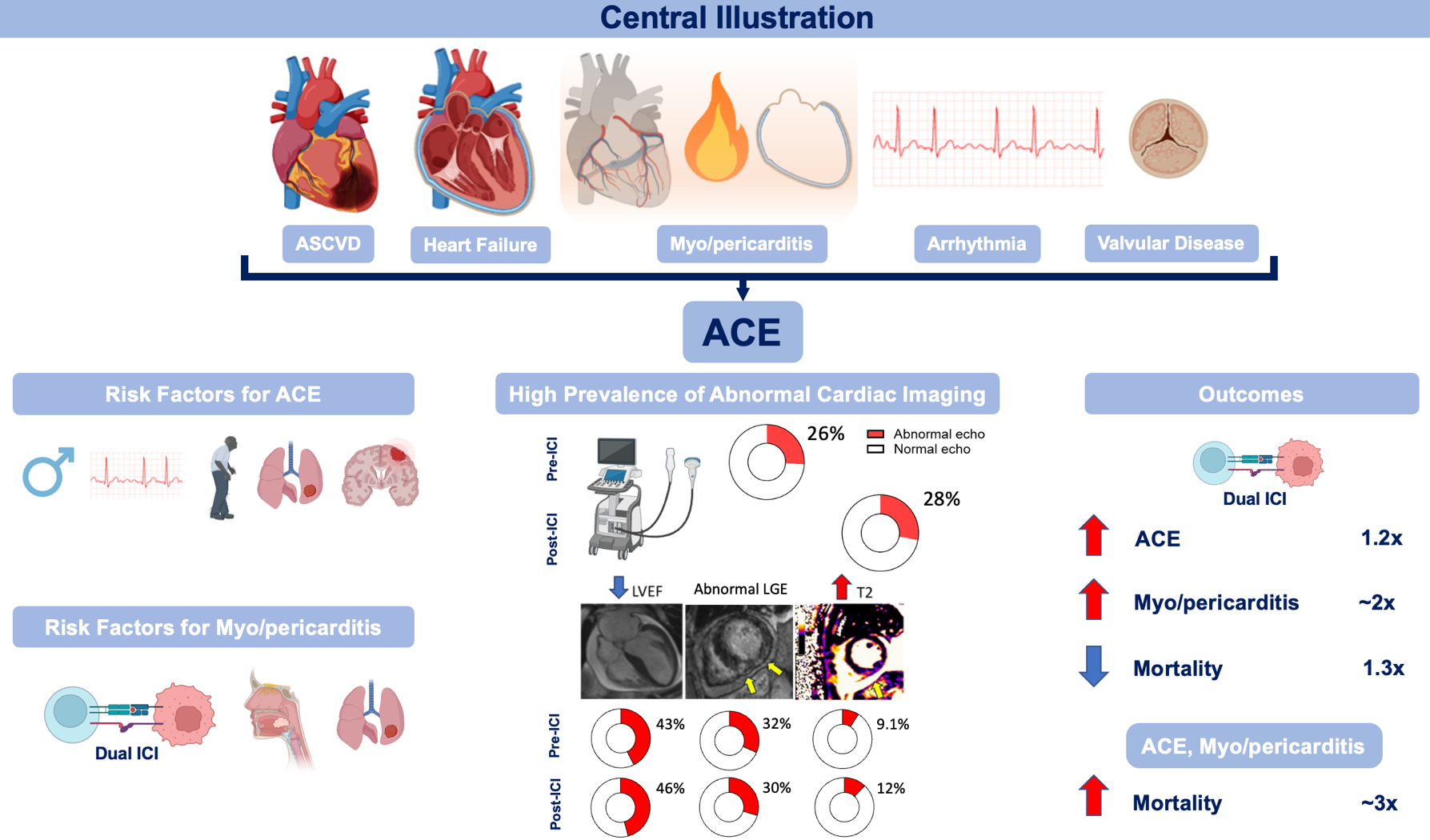

Supplement: S1 File — (DOCX) [file pone.0314555.s011.docx]
