# Supplementary material for: Adverse cardiovascular events and cardiac imaging findings in patients on immune checkpoint inhibitors
Source: PLoS One. 2024 Dec 2;19(12):e0314555. doi: 10.1371/journal.pone.0314555 (PMC11611253; doi:10.1371/journal.pone.0314555)
Supplement: Attachment — Submitted filename: Response to Reviewers.docx [file pone.0314555.s012.docx]

October 30, 2024

Sai-Ching Jim Yeung, MD, PhD

Academic Editor

PLOS ONE

Dear Dr. Yeung,

Re: Resubmission of manuscript PONE-D-24-31391 titled ‘*Adverse Cardiovascular Events and Cardiac Imaging Findings in Patients on Immune Checkpoint Inhibitors*’ for publication in *PLOS ONE*.

Thank you so much for the opportunity to revise our manuscript – we are greatly appreciative of the careful review and constructive feedback that we have received from the reviewers. Our point-by-point responses to reviewer comments are given on the following pages. We have made considerable edits, which have improved upon the quality of our manuscript.

We believe that the revised manuscript is now suitable for publication and we look forward to hearing from you soon.

Sincerely,

Lauren A. Baldassarre, MD, FACC, FSCMR, FSCCT

Associate Professor of Cardiovascular Medicine and Radiology & Biomedical Imaging

Vice Chief, Section of Cardiovascular Medicine, Faculty Life

Cardiology Director, Cardiac MR/CT

Director, Cardio-Oncology Program and Fellowship

Yale School of Medicine

**RESPONSE TO REVIEWER COMMENTS**

**Reviewer #1:**

This is a well written manuscript.

I wonder whether it is possible to develop a computer program based on your data and research which can calculate the pre-treatment risk and survival chance of an individual patient. This calculation could be similar to the heart score for chest pain with the calculation of a heart score which assigns a low risk, medium risk and high risk to the patient. The risk factors could include dual versus single use of ICI, age, sex, arrhythmia, lung cancer and CNS cancer. A score can certainly not predict the exact course of he ICI treatment but it could be helpful in the discussion with the patient searching for answers about treatment, probability of adverse effects of treatment and prognosis for treatment with ICI.

A newly developed score which can be computer generated could assist o find answers for the patient and the treating physicians when giving informed consent for ICI treatment. Additionally, table 2 A can be helpful to explain the risks of treatment and the fact that these adverse effects must be weighted towards the increased survival time with ICIs.

Maybe you can mention that based on your research you could try to develop such a computer program to calculate an ICI treatment score for risk stratification for adverse cardiac events within a certain period of time.

**Response:** We thank the reviewer for their thoughtful and supportive comments. The reviewer’s suggestion about implementation of a computerized model for risk stratification is a great suggestion. We are in the process of expanding our cohort with more current data, which will eventually allow us to generate a robust risk model. We have added a segment under the “Conclusion” section, highlighting our plan to develop and implement a risk model as a future direction.

**Reviewer #2:**

SMOKING is a primary risk factor for ACE and represents a critical source of potential confounding in this study. Its absence from basic demographics of the cohort, as well as its absence in the propensity score matching, represents a significant flaw in this study. Any information on smoking status (previous, never, etc) is better than nothing to better understand how it fits in with pre/post ICI associative data.

Overall, this paper appears to emulate Drobni et al 2020, but does so with worse methodology (retrospective cohort rather than case-control, an even larger composite outcome, and with significant potential confounders that aren’t addressed with their propensity matching), and I think these issues should be addressed, if possible, with data re-analysis prior to publication (ex: data on previous cardiotoxic cancer therapies, and smoking status). I think this study adds a large body of data to support relevant associations such as dual ICI with increased risk ACE, mortality, and myo/pericarditis; there is thought-provoking data suggesting a not insignificant mortality benefit association from dual ICI (despite apparent associated risk of ACE/myo/pericarditis). It could still be worthy of publication but the data needs to be presented in a clearer way with less conflicting information. See below for line-by-line comments. Also, I am definitely not a statistician, so please excuse any errors I may have made and help me understand further if I have made mistakes. I wish you luck in improving your paper and hope these comments help get you closer to publication.

**Response:** We thank the reviewer for their constructive feedback. We agree that smoking history is an incredibly important cardiovascular risk factor that should have been included – per the reviewer’s suggestion, we have reanalyzed our data to include smoking status (i.e. ever vs never smoker) and revised the manuscript text, tables, and figures to include this information. There were marginal changes in hazard ratios and confidence intervals of other covariates with the inclusion of the smoking data, and these changes are reflected in the manuscript text. There were no changes in which covariates were considered statistically significant for any outcome. In Table 1, there was no significant difference in smoking status between the ACE and no ACE groups (*P* = 0.309). In our multivariable analysis (Figure 1), smoking was not significantly associated with an increased risk of composite ACE or the individual components of ACE; however, it was most strongly associated with an increased risk of ASCVD (HR 1.22; CI 1.00-1.48), but this barely did not meet significance with a P=0.052. Similarly, in the multivariable analysis including single immune checkpoint inhibitor agents for ACE (Supplemental Figure 3), smoking was not significantly associated with increased risk of composite ACE or individual components of ACE, although again this trended towards significance for ASCVD.

We must respectfully disagree with the reviewer’s statement regarding their comparison with the study by Drobni et al 2020. We re-reviewed the paper by Drobni et al and it is considered a retrospective matched cohort study, not a case-control study. Retrospective cohort studies compare the risk of developing a particular disease state after known exposures (e.g. ICI use), whereas a case-control studies attempt to retroactively determine possible exposures after first identifying a known disease state. As such, the methodology in the paper by Drobni et al is fundamentally similar to ours, with the exception of matching methodologies. In Drobni et al, investigators performed 1:1 matching on the basis of age, history of cardiovascular agents, and cancer type, utilizing cancer patients from a different study period who did not receive ICI. In contrast, our study performed propensity matching utilizing cancer patients from the *same* study period who received TKIs, which do not classically carry a class effect of cardiotoxicity, unlike other agents such as anthracyclines, which carry an established class effect of cardiotoxicity. In contrast to study by Drobni et al, our study provides additional granularity into the broad spectrum of ACE as well as the outcome of myo/pericarditis and lends additional credence to the importance of pertinent risk factors such as dual ICI use. In contrast to the imaging substudy from Drobni et al, which evaluated aortic plaque burden via CT, our imaging substudy investigates vastly different cardiac imaging modalities (I.e. echocardiography and cardiac MRI); both echocardiography and cardiac MRI provide valuable insights into cardiac physiology and pathology. Moreover, the added value of tissue characterization with cardiac MRI is a profoundly unique aspect of this study.

ABSTRACT:

Line 49: CNS malignancy is included as independently associated with ACE, however Supplemental Figure 3 seems to show “neuro” as crossing 1.0 in the top panel. Why do much of the data in that cox regression analysis in Supp. Fig. 3 conflict with the primary Figure 1 results? Is the primary difference that Supp. Fig. 3 included individual ICIs in the analysis? It would be helpful if the panels were formatted in the same orientation as Figure 1 so it makes it easier to compare differences. Overall, I’m not seeing the face validity of CNS malignancies being independently associated with ACE, and the presence of multiple conflicting points in the large amount of data graphics presented is a problem (ex: Figure 1 v. Supp. Fig. 3; “ACE” panel in Supp. Fig. 3 shows “neuro” crossing 1.0 suggesting statistical insignificance, yet Figure 1 shows “CNS malignancy” as statistically significantly associated with increased ACE; or “Arrhythmia” panel in Supp. Fig. 3. Shows “neuro” as actually protective from arrhythmias, yet Figure 1 shows “CNS malignancy” trending toward protective but ultimately crossing 1.0 – which is ironic because Line 318 in the Discussion section offers that “arrhythmias” may contribute to higher rates of ACE, despite the data not supporting that hypothesis at all, at least I’m not seeing support in Figure 1 or Supp. Fig. 3). Perhaps I’m misunderstanding something, however, so please help me understand. Also, if “neuro” in the Supp. Fig 3 is meant to be the same as “CNS malignancy” in Figure 1, then please edit the labels and reorient the panels so they appears as similar to Figure 1 as possible to make it easier to compare the two sets of Cox Regression Models.

**Response:** The differences between Figure 1 and Supplemental Figure 3 stem from inherent differences in covariate selection resulting in different multivariable models. In Supplemental Figure 3, single ICI agents were included as covariates in order to investigate whether specific ICI agents were independently associated with composite ACE or individual ACE outcomes, which they weren’t. In contrast, Figure 1 is based on ICI all-comers and does not include individual ICI agents as covariates. Despite differences in statistical significance between the two models, the hazard ratios for the various covariates largely trend in the same direction. Although CNS malignancy was independently associated with ACE in Figure 1 but not significantly associated with ACE in Supplemental Figure 3, they do trend in the same direction. This perhaps suggests that dual ICI use contributes to a higher degree of toxicity. We added a segment in the results section addressing these differences. Per the reviewer’s suggestion, the formatting and labeling in Supplemental Figure 3 was updated to match the formatting in Figure 1 in order to facilitate comparison.

Line 54: “19 of 90 who had CMR imaging…” Where did n=90 come from? Supplemental Table 4 shows ordering indications and suggests a total of 102 pre-ICI CMRs and 28 post-ICI CMRs… yet the supplemental Table 5 lists Pre-ICI total as n=90 and post-ICI CMRs as 14. Why is there a discrepancy between these numbers? What happened to the other 26 CMR scans?

**Response:** Supplemental Table 4, which shows ordering indications, is not reflective of the number of scans, as there can be multiple ordering indications per CMR exam. Please refer to Supplemental Table 5 for the actual numbers. However, we do apologize for some inaccuracies in the Supplemental Table 5, which have since been corrected. In Supplemental Table 5B, the columns should be “pre-ICI” scans (n=21) and “post-ICI” scans (n=83). In Supplemental Table 5C, the columns should be “No ICI myocarditis” (n=90) and “ICI myocarditis” (n=14). The corresponding manuscript text has been updated to reflect these corrections.

Line 61: Again, “CNS malignancies” being included here needs to be supported by all of the data presented, and apparent conflict between Supp. Fig. 3 (“neuro”) and main Figure 1 (“CNS malignancies”) tables need to be explained.

**Response:** We apologize for the inconsistencies across figures. We have relabeled “neuro” as “CNS malignancies”. Please refer to the above response explaining the differences between Figure 1 and Supplemental Figure 3.

MAIN PAPER:

Line 117: “…prior to and after ICI administration.” – this statement seems pretty misleading considering my comment below regarding Lines 265-270.

**Response:** Please see responses below addressing the reviewer’s comments regarding lines 265-270. A statement has been added to the “Study population and covariates of interest” section, stating that “cardiac imaging was obtained when clinically indicated, and all pre-ICI scans were compared with post-ICI scans.” Subsequent verbiage throughout the manuscript text has been adjusted.

Line 124-125: Was the "Joint Data Analytics Team" (data abstractor) blinded to study outcome measures? Could a supplemental document noting specific ICD code/variable requests be made available?

**Response:** Joint Data analytics team is an institution-wide service that supports multiple research groups and projects. No members of JDAT were involved in the study design, statistical analysis, and manuscript preparation. We provide a list of diagnoses and ICD codes to the Joint Data Analytics Team, which then pulls these variables of interest from the EMR. Subsequent analyses were performed by our own study investigators to assess outcomes of interest. We added a sentence in the “Methods” section stating the above. A supplemental document noting specific ICD codes could be made available upon request.

Line 149-150: Why was “pericardial effusion” not a component of abnormal echo? To my knowledge, it’s part of the diagnostic criterion for pericarditis, which is one of the primary outcomes being examined in both Figure 1 and Table 1. I imagine there are a great deal of clinically insignificant pericardial effusions, however if they’re universally present (or absent) in cases of confirmed ICI-related myo/pericarditis then I think it’s work reporting. Why was “valvular disease” not a component of abnormal echo? It’s one of the primary outcome elements (“valvular disease [moderate or severe]”).

**Response:** We agree that evaluating for pericardial effusions, which could be a manifestation of toxicity in the setting of ICI-related pericarditis, is a relevant parameter to look into. However, out of all patients with echocardiograms, only 3 out of the entire cohort had at least a moderately-sized pericardial effusion, limiting our ability to make any meaningful statistical inferences. Regarding your comment about valvular disease, we feel that the assessment of valvular disease is less relevant for our study, as ICIs are not known to be associated with worsening valvular disease, although this is an interesting question for a follow-up study.

Line 171-172: I think a little more detail regarding coding techniques/keywords/instructions involved in using NLP in this way would be appreciated.

**Response**: Standard coding methodologies were utilized for the NLTK toolkit in constructing the NLP pipeline. In-depth techniques on the use of NLTK are featured in this book: Bird, Steven, Edward Loper and Ewan Klein (2009), Natural Language Processing with Python. O’Reilly Media Inc. We have cited this resource within the manuscript text with a reference added to the references section. Our code could be made available upon reasonable request.

Line 192-193: In my anecdotal experience, I see ipilimumab being utilized more often as part of a dual ICI strategy (ex: ipi/nivo) than as a mono-ICI. That’s in contrast to frequently seeing nivolumab monotherapy (for whatever reason). Does the data in Table 2 isolate instances of ipilimumab as only monotherapy, or could recorded instances of its use for dual ICI be potentially driving its appearance as an outlier in that list?

**Response:** You are correct that we often see ipi/nivo used in combination. The data in Table 2 does not isolate instances of ipilimumab as only monotherapy. We did not specify which dual agents were used, and this was not one of our study objectives. We found that dual ICI use, irrespective of which specific agents were used, was associated with increased risk of composite ACE and myo/pericarditis. Future studies can be conducted to assess for any potential differences in the various combinations of dual ICI therapy.

Line 199: Why are the “n” values so large and varied in this list? It would appear obviously much larger than total cohort of 2165. Please help me understand where these total values are coming from.

**Response:** The cumulative n values are larger than the cohort of 2165 because patients can have multiple malignancies.

Line 210-212: How is prior ASCVD associated with a lower risk of ASCVD following ICI therapy? This seems completely counterintuitive and potentially a data error of some kind. This requires further explanation.

**Response:** One possible explanation for this observation is that patients with prior ASCVD have been established with cardiologists/cardio-oncologists and have been initiated on cardioprotective agents, leading to lower risk of developing future ASCVD events, as opposed to those who have undiagnosed ASCVD who may not yet be on optimal medical therapy. However, this would need to be further evaluated in a prospective study.

Line 227: see my first comment above (Line 49) that has concerns regarding Supplemental Figure 3.

**Response:** See above response.

Line 228-230: “after adjusting for covariates in a multivariable Cox regression model, it was not independently associated with…” need to add a bolded reference to Supplemental Figure 3 at the end of this sentence.

**Response:** Done.

Line 237-238: “However, dual ICI therapy was found to decrease mortality by ~25%.” I see in line 299-305 the authors suggest that low incidence of myocarditis suggests “the majority of patients likely still [derive] mortality benefit from ICI therapy.” – if this is correct, then perhaps this findings is worthy of inclusion in the abstract for the paper.

**Response**: We agree with this statement and have highlighted this finding in the abstract.

Line 249: Supplemental Table 3 – please edit the table so the variable presented match Table 1 (ex: either switch Table 1 to be alphabetical, or re-order Supplemental Table 3 to more closely match the order of variables presented in Table 1).

**Response:** Done.

Line 258: “Supplemental Figure 4” – please add “n” information to the infographic to clearly see how many echo exams were performed.

**Response:** Done.

Line 265: Why does Supplemental Table 4 indicate a total of 130 CMR exams (102 pre-ICI, 28 post-ICI) that were ordered? Were 26 CMR studies ordered but never completed? Perhaps add bolded reference to Supplemental Table 5 at the end of this sentence on line 268.

**Response:** There can be multiple indications entered when ordering CMR, which is why the numbers don’t exactly add up.

Line 265: “(5%)” perhaps add clarification of some kind such as “104 of the entire cohort of 2165 (5%) had CMR performed…”

**Response**: Done.

Line 265: “21 (20%) of CMR imaging being performed prior to ICI initiation…” – Supp. Table 5 suggests 90/104 (87%) were performed prior to ICI initiation. Why the discrepancy?

**Response:** We apologize for the confusion. There were inaccuracies in the Supplemental Table 5, which have been corrected. In Supplemental Table 5B, the columns should be “pre-ICI” scans (n=21) and “post-ICI” scans (n=83). In Supplemental Table 5C, the columns should be “No ICI myocarditis” (n=90) and “ICI myocarditis” (n=14). The corresponding manuscript text has been updated to reflect these corrections.

Line 267: “…83 (80%) of CMR images being performed after ICI initiation…” – Supp. Table 5 suggests 14/104 (13%) were performed post-ICI initiation. Why the discrepancy?

**Response:** Please see above response.

Line 268-270: If only 4 patients had CMR imaging both pre/post ICI and only 1 had diagnostic value… perhaps the entire “substudy” portion involving CMR imaging should be re-framed to focus solely on the increased LGE post-ICI (one of the only parts of Supp. Table 5 to have proper significance). I’m not familiar with “RV Insertion LGE” but that appeared to have p<0.05 in Supp. Table 5.

**Response:** After making corrections to Supplemental Table 5, there are no longer significant differences in CMR characteristics pre- and post-ICI. We have thus shifted our focus to highlight the prevalence of abnormal CMR characteristics prior to ICI initiation, which is an important finding that highlights the need for aggressive cardiovascular disease prevention and risk factor modification in this high-risk population. Regarding the finding of RV insertion LGE, this is somewhat of a non-specific finding that can be present in various cardiomyopathies and pulmonary hypertension; nevertheless, we report this finding if observed.

Line 273: “Supplemental Table 5B” – Abnormal T1 findings are part of the “Lake Louise Criteria” referenced in Line 113, how come that variable is absent in this table?

**Response:** The presence of late gadolinium enhancement is considered a form of abnormal T1. Manuscript text has been revised to make this clearer.

Line 277: Supp. Table 5 indicates T2 findings did not achieve statistical significance (P=0.063), perhaps add a qualifying statement to this sentence explicitly stating lack of p-value significance.

**Response:** Done.

Line 287: Central Illustration – “Outcomes” in the bottom right portion of the figure – is there a way to keep the units equivalent when comparing the relative pros/cons of dual ICI? It’s tough to compare “~2x” or “~3x” with “25%.”

**Response:** A ~25% decrease in mortality would correspond to a 1.3x fold decrease in mortality. The manuscript text and central illustration have been modified accordingly.

Line 312-315: this is a huge limitation, and calls to question the validity of the propensity score matching in this dataset.

**Response:** We have addressed this limitation through the reanalysis of our data to include smoking status.

Line 350-351: why wasn’t troponin or BNP included in this dataset?

**Response:** Our Joint Data Analytics Team attempted to pull troponin data from the EHR but unfortunately ran into technical issues, which was why this was not included in the dataset.

Line 364: the absence of “smoking” and “previous cardiotoxic treatments” suggests it wasn’t adjusted as much as possible. Surely some information on these confounders are extractable from the EHR.

**Response:** As suggested, we have updated our analysis with smoking status. Please see the above response.

Line 365: “…with echo and CMR obtained prior to and after ICI initiation” – again, somewhat misleading verbiage, please find a way to word this to clearly state cardiac imaging was obtained when clinically indicated, and all pre-ICI scans were compared with post-ICI scans, though almost none of them were in the same patient.

**Response:** Thank you for this constructive feedback. We have added a sentence to the end of the “Study population and covariates of interest” section to provide more accurate verbiage. We have also added a segment in the “Study Limitations” section, mentioning that almost no pre- and post-ICI scans were in the same patient. This is certainly an area of interest that we will explore further in a future study.

Line 372: consider adding that “ACE” is a composite outcome with composite elements (ex: ASCVD) above and beyond what is typically considered “MACE.” This will probably make this data very difficult to

**Response:** Sorry, we do not understand the reviewer’s question given that it was not completed. We defined the definition of ACE in our study under “Study Outcomes” which was a “composite of ASCVD — which included coronary artery disease (CAD), myocardial infarction (MI), stroke (CVA), and peripheral arterial disease (PAD) —, heart failure (HF), arrhythmia (includes supraventricular tachycardia, atrial fibrillation, atrial flutter, ventricular tachycardia, ventricular fibrillation)), valvular disease (moderate or severe), pericardial disease, and myocarditis after initiation of ICI therapy.”

Line 377: is 10% individual chart review good enough? Is there a way to demonstrate that ~200 charts of 2000 is sufficiently powered to make the conclusion that there are no significant differences between the EHR data pull and chart review?

**Response:** The concept of powering is not applicable to our study design, given that we are not attempting to perform a clinical trial. The fact that we were able to perform a manual chart adjudication is already a study strength given that many retrospective studies that utilize large datasets from broad EHR data pulls do not perform manual adjudication.

Line 392: if “metastatic disease” is highlighted in the main body of the conclusion, I would recommend highlighting that in the abstract rather than “CNS malignancy.”

**Response:** We have revised the main body of the conclusion to highlight CNS malignancy instead of metastatic disease.
